# Clinical, Laboratory, and Therapeutic Aspects of* Crotalus durissus* (South American Rattlesnake) Victims: A Literature Review

**DOI:** 10.1155/2019/1345923

**Published:** 2019-08-05

**Authors:** Bruno Tumang Frare, Yann Keller Silva Resende, Bruno de Carvalho Dornelas, Miguel Tannus Jorge, Valter Augusto Souza Ricarte, Lívia Maria Alves, Luiz Fernando Moreira Izidoro

**Affiliations:** ^1^Faculty of Medicine, Universidade Federal de Uberlândia, Minas Gerais, Brazil; ^2^Institute of Biomedical Science, Universidade Federal de Uberlândia, Minas Gerais, Brazil; ^3^Institute of Biotecnology, Universidade Federal de Uberlândia, Minas Gerais, Brazil

## Abstract

Snakebite envenoming is a neglected public health issue in many tropical and subtropical countries. To diagnosis and treat snakebites may be challenging to health care personnel since sufficient information has not been yet provided. This review presents the clinical, therapeutic, and laboratory aspects of* Crotalus durissus* (South American rattlesnakes) victims. The clinical setting may show local effects such as little or no pain, mild edema, and recurrent erythema. In contrast, the systemic effects may be quite remarkable, such as changes due to neurological damage, intense rhabdomyolysis, incoagulability of the blood, and variations in the peripheral blood elements. The main complication is acute kidney injury. The appropriate treatment depends mainly on the correct recognition of the aggressor snake and the symptoms expressed by the victim. Rattlesnake venom can cause irreparable damage and lead to death. Therefore, a prompt diagnosis allows the immediate onset of proper serotherapy.

## 1. Introduction

Snakebite envenoming results of the injection of a highly specialized toxic secretion by a venomous snake; usually it occurs under accidental circumstances [1]. About 5.4 million snakebites happen a year, resulting in 1.8 to 2.7 million cases of envenoming. There are between 81,410 and 137,880 deaths and around three times fold as many amputations and other permanent disabilities each year [2]. In a recent study carried on by Chippaux et al. [3], about 60,000 snakebites were reported annually in health facilities in the Americas, and approximately 370 cases evolve to death. Specifically in Brazil, venomous snakes are encountered in all territory, and the occurrences show an exacerbated lethality in areas with limited access to healthcare assistance. Between 2000 and 2017, there were 471,801 cases of ophidian envenomation in the country, and 1,892 deaths were reported. The highest incidence occurred in the North Region of Brazil, where 142,230 cases and 647 deaths were notified [4].

Geographically, the greatest impact of snakebites hits the tropical and subtropical regions, due to a combination of factors. Snake density and developing-world status imply in limited access footwear, flashlights, and mostly adequate medical care [5]. Globally, poverty is a distinctive characteristic of the neglected tropical diseases [6]. Recognized as a serious health problem in tropical regions, snakebite envenoming has been reinserted on the list of neglected tropical diseases in 2017 by the World Health Organization (WHO), after being removed in 2013 [7–9].

Until now, the antivenoms are the only specific treatment for snakebite envenoming [9]. Antivenoms are composed of entire or enzyme-digested equine or ovine immunoglobulins which can neutralize the circulating venom toxins specifically [10]. An appropriate antivenom must be selected based on identification of the snake specie [1]. Antivenoms are costly and often scarce. The availability and accessibility are not uniform among the most vulnerable populations worldwide [1, 9, 11].

Another important point is the establishment of treatment which can be difficult to inexperienced physicians or not specialized healthcare personnel. In an insufficient information scenario, a lack of knowledge regarding signs of envenoming, time of the accidental bite, snake genus, amount of injected venom, site of injection, and patient's health status may impair the appropriate management. A delay in treating is corroborated by current clinical guidelines which recommend withholding antivenom administration until envenoming systemic symptoms are detected [1, 12].

This review presents the clinical, therapeutic and laboratory aspects of* Crotalus durissus* (South American rattlesnakes) victims, as well as the relationship in-between the crotalic venom composition and the snakebite manifestations.

## 2. *Crotalus durissus* Snakes

The Elapidae and Viperidae are by far the most important snake families out of the five ones capable of envenoming humans [10]. In Brazil, envenoming snakes are represented by the genera* Bothrops*,* Crotalus*,* Lachesis, *and* Micrurus* [13]. The* Crotalus* genus belongs to the Viperidae family and includes approximately 70 species and subspecies known as rattlesnakes [14]. These species are found throughout the American continent from southern Canada to the central region of Argentina [15]. In Brazil, the unique specie* Crotalus durissus*, commonly known as rattlesnake, is subdivided into seven subspecies:* C. durissus dryinas* (Amapá State),* C. durissus terrificus* (South Region),* C. durissus cascavella* (Northeast Region),* C. durissus trigonicus* (Roraima State),* C. durissus ruruima* (North Region),* C. durissus marajoensis* (Marajó Island), and* C. durissus collilineatus *(Midwest Region) [16].

*C. durissus* snakes are robust and may reach one meter in length. Its most notable feature is a rattle at the end of the tail which simplifies the recognition. These snakes can be seen in open fields and dry, sandy, and stony areas. They are rarely fund in coastal and marshy regions [17, 18].* C. durissus* subspecies envenoming are usually severe and frequently fatal in the absence of adequate treatment. The accident incidence shows seasonality, mostly occurring from October to April [19]. According to data published by Brazil's National Disease Notification System (SINAN) in 2017, the* Crotalus* genus was responsible for 2,503 cases with a lethality rate of 0.71% [20].

## 3. Crotalic Venom

Some animals produce venoms to defend themselves from predators or to obtain food. Snake venoms are a mixture of mostly enzymes and nonenzymatic proteins/peptides used for immobilization, killing, and digestion of preys [21]. The consequences to the victim depend on the toxin composition which correlates with the clinical manifestation and pathological effects such as neurotoxicity, systemic myonecrosis, hemostatic disorders, myoglobinuria, and acute renal failure [18, 22].

The venom composition and the clinical and laboratory aspects caused by* C. durissus terrificus* and* C. durissus collilineatus* ophidism are the most studied ones [16, 22–24]. The general pharmacology and composition of the venom from the various* Crotalus* species in Brazil are very similar. There are some intraspecific and interspecific variabilities but they all are essentially composed of four toxins: crotoxin, crotamine, gyroxin, and convulxin [16, 23, 25, 26].

Crotoxin is the major toxic component of the South American rattlesnake* C. durissus *venom. It is constituted of two different subunits, a weak toxic basic A2 phospholipase with high enzymatic activity, and an acidic polypeptide devoid of toxic and enzymatic activities, called crotapotin [27]. The severe neurotoxic symptoms and acute nephrotoxicity are attributable to the high amounts of the crotoxin [26]. Crotamine is a low molecular weight, nonenzymatic, and noncytolytic small protein composed of about 42 amino acid residues. It is one of the major components of* C. durissus terrificus* venom that combines the cytotoxic and neurotoxic properties [28]. Gyroxin is a serine protease that accounts for about 2% of the total protein content of the venom. It induces hemotoxicity and a neurological barrel rotation syndrome in mice [22, 29]. Convulxin is a C-type lectin, a potent platelet-aggregating glycoprotein, without any enzymatic actions. It consists of two subunits (alpha and beta) linked by disulfide bridges in a hexameric structure and causes cardiovascular and respiratory disturbs [22, 30, 31].

## 4. Clinical and Laboratory Aspects

Snakebite envenomings are a medical emergency due to their potential rapid lethality [1]. In many cases, the snake responsible for the accident remains unidentified what frequently makes it difficult to decide which antivenom to be administered to the victim, especially when only monospecific antivenoms are available [32].

Laboratory confirmation may be made by crotalic venom antigens which can be detected in the blood or other patient's body fluids by ELISA technique [17].

The diagnosis of snakebite or determination of which snake is responsible for envenoming of a victim can be conveniently divided into clinical and laboratory diagnosis. Clinical diagnosis depends upon recognizing specific signs of envenoming in the patient that includes local signs such as swelling, blistering, and local necrosis and systemic signs, such as hemorrhage, incoagulable blood, and hypovolaemic shock, neurotoxic signs, and rhabdomyolysis. Laboratory diagnosis includes the detection of abnormal changes in blood parameters (blood clotting test and dramatic fall in the platelet count, changes in red and white blood cell counts), presence/absence of myoglobinuria, changes in certain enzyme levels (such as creatine kinase), and the detection in the blood of the victims and others [32].

Based on clinical manifestations, crotalic envenoming may be classified as mild, moderate, and severe. Mild envenoming characterized by the presence of discrete neurotoxic signs and symptoms, without myalgia or altered urine color or mild myalgia; the moderate is characterized by the presence of discrete neurotoxic signs and symptoms, discrete myalgia and myoglobinuria; and severe, where neurotoxic signs and symptoms are evident and intense (myasthenic facies; muscle weakness), myalgia is intense and widespread, urine is dark, and there may be oliguria or anuria [33].

Local manifestations include usually mild or absent pain and edema, with erythema and paresthesia [33–35]. Neurological systemic manifestations appear in the first hours after the bite, and characterize the myasthenic facies (Rosenfeld neurotoxic facies) evidenced by uni- or bilateral palpebral ptosis, facial muscle flaccidity, pupillary diameter alteration, ophthalmoplegia, blurred vision, and/or diplopia [13, 34–36]. Other manifestations, such as difficulty in swallowing, velopalatine paralysis, increased vomiting reflex, and taste and smell alterations may also be observed in those more severe cases [17].

Myotoxic activity produces systemic skeletal muscle lesions (rhabdomyolysis), leading to the release of enzymes and myoglobin into the blood, which are then excreted in the urine. The clinical characterization of this venom action is the appearance of dark urine due to the elimination of variable amounts of myoglobin (myoglobinuria) and generalized muscular pain (myalgia), which are more intense in the more severe conditions [13, 37]. The myotoxic activity of the venom of* C. durissus terrificus* is demonstrable by increased serum levels of the enzymes creatine kinase (CK), lactate dehydrogenase (LDH), and aspartate aminotransferase (AST) [38]. CK increase is early, with peak maximum elevation within the first 24 hours after the accident. The increase in LDH is slow and gradual and therefore constitutes an examination for late diagnosis of crotalic envenoming [17].

Extensive and severe myocardial lesions characterized by sarcoplasmic vacuoles, densely clumped myofibrils, and amorphous acidophilic mass into the cardiac fibers were reported in a patient who died due to snakebite by* C durissus terrificus* [39]. However, Cupo et al. [40] and Cupo et al. [38] showed that although the enzyme release pattern was similar to acute myocardial infarction, the results of the electrocardiogram and echocardiogram examinations did not demonstrate cardiac muscle involvement, raising the hypothesis that crotalic venom causes preferential damage in muscle fibers of type I and/or type IIa, rich in the enzymes CK-MB and LD1, and similar to those found in the myocardium. Other relevant information that rules out cardiac damage was normal plasma troponin I [40].

The blood count may show leukocytosis, neutrophilia, lymphopenia and even the occasional presence of blast cells in the peripheral blood. These changes also occur in the first hours after poisoning. After 72 hours, the presence of eosinophils may be striking, while the other cell types such as monocytes, lymphocytes, and basophils do not experience absolute variations [36].

Envenoming by* C. durissus* is frequently associated with hemostatic disorders, which are probably attributable mainly to the action of the thrombin-like enzyme, with possible additional effects secondary to the powerful myotoxic activity of the venom [41]. Duarte and collaborators [42] use thrombin generation test to evaluate in vitro hemostatic changes caused by the venom of Brazilian snakes* Bothrops jararacussu*,* Bothrops alternatus*,* Bothrops moojeni*, and* Crotalus durissus terrificus* in a normal pool of platelet-poor plasma and the genus* Crotalus *venom showed less procoagulant activity in vitro compared to the others.

Several hematological changes that worsen the clinical status of the victim can be observed in the first hours after the bite. Commonly, there is a depletion of some factors of the coagulation cascade that leads, for example, to afibrinogenemia, hypofibrinogenemia, and consequent prolongation of prothrombin time and activated partial thromboplastin time [43]. There may be blood incoagulability or increased coagulation time and rarely gingival bleeding [36].

Due to their complex composition, venoms act through various mechanisms in the victim, affecting also the physiology of the hepatic tissue. Barravieira et al. [44] observed hepatic dysfunction in a patient after an intraocular accident with* C. durissus.* In this study, one of the patients evolved to death and the anatomopathological examination of the liver presented a hydropic degeneration and mitochondrial lesions. Silva et al. [45] investigated the effects of the* C. durissus terrificus* venom in the liver of experimental animals (Wistar rats), the plasma activities of alanine aminotransferase (ALT), and aspartate aminotransferase and hepatic glutathione S-transferase and catalase presented significant elevation and the results demonstrated that the venom can produce several changes in hepatocytes and the authors suggest that the causes of the changes are possibly related to the disequilibrium in the redox homeostasis but also specific needs of the poisoned organism, for example, an increased supply of lactate and pyruvate in response to an increased activity of the Cori cycle.

One of the main complications of accidents caused by* Crotalus* snakes is an acute renal failure and the clinical manifestation of ARF may occur in the first 48 hours after the accident [36]. Risk factors for ARF development include time interval for antivenom administration, rhabdomyolysis, patient age, and urinary volume [18, 46]. Myalgia and neurotoxic facies were predictive of renal failure in patients older than 40 years [47]. Diverse mechanisms have been proposed to explain this injury, including rhabdomyolysis, hemolysis, shock, intravascular coagulation, and a possible direct nephrotoxic effect; once the venom is excreted by the kidneys, the mechanisms of concentration and tubular transport favor the occurrence of direct cellular toxicity [48].

Snakebites by* C. durissus *in children in Campinas, São Paulo, Brazil were described. Of 31 children under 15 old admitted, one patient was classified as “dry-bite”, 3 as mild envenoming, 9 as moderate envenoming, and 18 as severe envenoming. Most patients had neuromuscular manifestations, such as palpebral ptosis, myalgia, and weakness. Laboratory tests suggesting rhabdomyolysis included an increase in total blood CK and LDH levels and myoglobinuria. The main local signs and symptoms were slight edema and erythema. Before antivenom administration, blood coagulation disorders were observed in 20/25 children (incoagulable blood in 17/25). Patients admitted less than and more than 6 h after the bite showed the same risk of developing severe envenoming. No children admitted less than 6 hours showed severe complications, while half of children admitted more than 6 h after bite developed acute renal failure; however, no deaths were recorded [49].

“Dry-bite” is a phenomenon that occurs when a venomous snake bites a person and the victim does not have any clinical signs, local or systemic, typical of envenomation. This accounts for a sizable proportion of venomous snake bites all over the world, but not possible to pinpoint the exact reasons for its occurrence [50]. During an observational study (36 months) on South American rattlesnake bites in Minas Gerais, Brazil, 12% of 41 patients with fang marks at the bite-site did not present clinical or laboratory features of envenoming and had no plasma venom detected before specific antivenom, fulfilling the criteria for the diagnosis of “dry-bite”, and these dates suggest that these patients should be correctly diagnosed since they should not be treated with unnecessary and sometimes hazardous and expensive antivenom [51]. Jorge et al. [52] also observed 8 cases (of a total of 97) of snakebite without envenoming presenting at the Hospital Vital Brazil, in São Paulo, Brazil.

There are also some isolated cases with atypical characteristics. Compartment syndrome is an unusual but severe complication of bites by crotaline snakes, including some North American rattlesnakes; Bucaretchi* et al*. [53] reported a case of a South American rattlesnake (*C. durissus terrificus*) envenomation whose subfascial venom injection culminates in increased intracompartmental pressure and acute compartment syndrome as a complication.

*C. durissus terrificus* venoms from newly captured snakes and already-captured animals (kept in captivity) from the Botucatu region (SP, Brazil) were characterized comparatively and the results demonstrated high variability in the concentrations and decreased toxic activity in animals (LD_50_) during the captivity period; however, the chromatographic profiles did not present variation of venom proteins when considering the captivity time [54]. Baum et al. [55] reported a case of envenomation to a professional herpetologist in the United States by a captive-born specimen of* C. durissus terrificus* of Paraguay lineage and resulted in respiratory failure and therapeutic improvement following antivenom administration. Laboratory parameters all remained within normal limits from the time of hospital admission through the time of discharge, except for CK and the patient did not experience any coagulopathy during the hospital course of stay. During a study conducted by Jorge et al. [34], 3 patients developed respiratory failure (of 249 cases analyzed). Oliveira Neto et al. [56] analyzed the pulmonary function in an experimental model of acute lung injury (in mice) induced by the* C. durissus cascavella* venom and the venom caused mechanical and histopathological changes in the lung tissue.

The difference between accidents caused by small and large South American rattlesnakes was evaluated. Most of the accidents observed were caused by large snakes and among the clinical manifestations; the only significant differences observed between the two groups were myalgia and dark urine, more common in the victims of large snakes, suggesting that these are more likely to cause rhabdomyolysis [52]. Lourenço* et al*. [54] compared the venom of adults and newborns in captivity* C. durissus terrificus* and newborns presented 60% protein lower than the proportion found in adults.

Rattlesnake bites in Brazil are generally caused by adult individuals, with most of the envenomed patients showing systemic manifestations that include varying degrees of neurotoxicity, rhabdomyolysis, and coagulopathy, with only mild or no local manifestations. Bucaretchi et al. [57] report a case of envenoming by a juvenile South American rattlesnake (*C. durissus terrificus*) that involved coagulopathy as the main systemic manifestation, without neuromyotoxic features (no remarkable local manifestations and no signs/symptoms of myasthenia or rhabdomyolysis and serum CK, LDH, ALT and AST within reference levels) normally associated with bites by adult specimens. Despite the delayed administration (5 days after snakebite), crotalic antivenom was effective in improving the blood coagulation.

## 5. Treatment and Prognosis

Specific antivenom is the only treatment to neutralize the toxicity of the venom and the precocity in applying the antivenom is crucial for the efficiency of the treatment [58]. Commercial equine antivenom specific for* C. durissus terrificus* venom is utilized for the treatment of victims bitten by all subspecies of rattlesnakes in Brazil [25]. Guidolin et al. [59] evaluated the efficiency of antivenom obtained from horses immunized with* C. durissus terrificus* crude venom and was able to recognize and neutralize not only the toxins in* C. durissus terrificus* but also the ones present in the venoms from* C. durissus collilineatus*,* C. durissus cascavella*, and* C. durissus marajoensis*.

In Brazil, the Butantan Institute, Ezequiel Dias Foundation, and Vital Brazil Institute are responsible for the production of these immune derivatives for the public health system [48], and the specific treatment for the crotalic accident is the intravenous infusion of anticrotalic serum or antibothropic-crotalic serum. According to data provided by [36], the dose of anticrotalic serum varies according to the severity of the case. The administration of 5 ampoules of antidote is recommended for mild cases; 10 ampoules are indicated for moderate cases and 20 ampoules for severe cases.

At the site of the bite, it is recommended to perform asepsis and analgesia, as pathogenic germs may be present in the mouth of the snake, the skin of the victim, and even contaminants at the bite wound. Tetanus prophylaxis should also be adopted [17], as should the use of common antiemetics for the treatment of nausea and vomiting [37].

The literature verifies that the late application of serum therapy is directly related to more severe clinical and laboratory manifestations, contributing especially to ARF [60]. Systemically, the focus of treatment is the kidney condition. Proper maintenance of hydration is of fundamental importance in the prevention of ARF; therefore, the urinary flow should be maintained between 1 ml and 2 ml/kg/h in children and between 30 and 40 ml/h in adults, contributing to the reversal of oliguria [36]. Associated with the hypercatabolic state, ARF is exacerbated due to the myoglobin supply to the renal tissue, which results in tubular necrosis. In this situation, it is recommended to minimize the damage caused by myoglobinuria through osmotic diuresis and urine alkalinization, as myoglobin precipitates at pH values lower than 6.5 [17]. It then becomes necessary to use hemodialysis to maintain the welfare of the victim [37].

The efficacy of treatment strategies is monitored through the general condition of the patient, taking into consideration clinical signs and symptoms and laboratory parameters routinely dosed. For mild and moderate clinical conditions and patients treated early, in the first six hours after the bite, total regression of clinical signs and symptoms is observed after a few days of treatment. For severe accidents and/or patients with late care, the prognosis is related to the existence of renal insufficiency/tubular necrosis, and the reversion of the clinical condition is extended for a longer time [36].

Literature data show the ineffectiveness of tourniquet to reduce the severity of* C. durissus* envenoming assessed through clinical and laboratory features and the occurrence of complications [61]. In the Brazilian Amazon, severe manifestations at admission delayed medical assistance, lack of antivenom administration, and ages ≥61 and 0-15 years were predictors of death in* C. durissus* snakebites [62].

## 6. Conclusions

In this review, we have provided information to clarify the clinical and laboratory diagnosis of crotalic snakebites. Even though laboratory analyses of crotalic accidents are simple to obtain, it is evident that professionals should be able to interpret these results in a critical and trustworthy way, relating the results to the clinical symptoms, diagnosis, treatment, and prognosis of the victims. Therefore, such information should be better implemented during the training of healthcare professionals, aiming to obtain a better qualification so that the clinical treatment is managed correctly, reducing the deleterious effects that currently culminate with a high lethality of crotalic accidents.
